# Short-term exposure to ambient air pollution and individual emergency department visits for COVID-19: a case-crossover study in Canada

**DOI:** 10.1136/thoraxjnl-2021-217602

**Published:** 2022-03-31

**Authors:** Eric Lavigne, Niilo Ryti, Antonio Gasparrini, Francesco Sera, Scott Weichenthal, Hong Chen, Teresa To, Greg J Evans, Liu Sun, Aman Dheri, Lionnel Lemogo, Serge Olivier Kotchi, Dave Stieb

**Affiliations:** 1 Air Sectors Assessment and Exposure Science Division, Health Canada, Ottawa, Ontario, Canada; 2 School of Epidemiology & Public Health, University of Ottawa, Ottawa, Ontario, Canada; 3 Center for Environmental and Respiratory Health Research (CERH), University of Oulu, Oulu, Finland; 4 Department of Public Health Environments and Society, London School of Hygiene & Tropical Medicine, London, UK; 5 Centre for Statistical Methodology, London School of Hygiene & Tropical Medicine, London, UK; 6 Centre on Climate Change and Planetary Health, London School of Hygiene & Tropical Medicine, London, UK; 7 Department of Statistics, Computer Science and Applications "G. Parenti", University of Florence, Florence, Italy; 8 Department of Epidemiology, Biostatistics, and Occupational Health, McGill University Montreal, Montreal, Quebec, Canada; 9 Dalla Lana School of Public Health, University of Toronto, Toronto, Ontario, Canada; 10 Environmental Health Science and Research Bureau, Health Canada, Ottawa, Ontario, Canada; 11 Public Health Ontario, Toronto, Ontario, Canada; 12 Institute for Clinical Evaluative Sciences, Toronto, Ontario, Canada; 13 Child Health Evaluative Sciences, The Hospital For Sick Children, Toronto, Ontario, Canada; 14 Department of Chemical Engineering, University of Toronto, Toronto, Ontario, Canada; 15 Environment and Climate Change Canada Montreal Office, Montreal, Ontario, Canada; 16 National Microbiology Laboratory, Public Health Agency of Canada, Saint-Hyacinthe, Ontario, Canada

**Keywords:** COVID-19

## Abstract

**Background:**

Ambient air pollution is thought to contribute to increased risk of COVID-19, but the evidence is controversial.

**Objective:**

To evaluate the associations between short-term variations in outdoor concentrations of ambient air pollution and COVID-19 emergency department (ED) visits.

**Methods:**

We conducted a case-crossover study of 78 255 COVID-19 ED visits in Alberta and Ontario, Canada between 1 March 2020 and 31 March 2021. Daily air pollution data (ie, fine particulate matter with diameter less than 2.5 µm (PM_2.5_), nitrogen dioxide (NO_2_) and ozone were assigned to individual case of COVID-19 in 10 km × 10 km grid resolution. Conditional logistic regression was used to estimate associations between air pollution and ED visits for COVID-19.

**Results:**

Cumulative ambient exposure over 0–3 days to PM2.5 (OR 1.010; 95% CI 1.004 to 1.015, per 6.2 µg/m^3^) and NO_2_ (OR 1.021; 95% CI 1.015 to 1.028, per 7.7 ppb) concentrations were associated with ED visits for COVID-19. We found that the association between PM_2.5_ and COVID-19 ED visits was stronger among those hospitalised following an ED visit, as a measure of disease severity, (OR 1.023; 95% CI 1.015 to 1.031) compared with those not hospitalised (OR 0.992; 95% CI 0.980 to 1.004) (p value for effect modification=0.04).

**Conclusions:**

We found associations between short-term exposure to ambient air pollutants and COVID-19 ED visits. Exposure to air pollution may also lead to more severe COVID-19 disease.

**Key messages**

**What is the key question?**

- Are short-term variations in outdoor concentrations of ambient air pollution associated with COVID-19 emergency department visits?

**What is the bottom line?**

- Acute COVID-19-related emergency department visits were associated with exposure to particulate matter with diameter less than 2.5 μm and nitrogen dioxide over the previous days before admission and exposure to air pollution may also lead to more severe COVID-19 disease.

**Why read on?**

- This is one of the very few studies assessing the association of interest using individual-level data on COVID-19 and using indicators of severity of COVID-19 disease.

## Introduction

Over the past decades, a large number of studies have shown that acute and chronic exposure to ambient air pollution is associated with increased morbidity and mortality.^1^ Globally, ambient air pollution is the leading environmental risk factor for deaths and disability based on estimates of the Global Burden of Disease initiative.^2^ Previous studies have shown associations between daily levels of ambient air pollution and acute pulmonary events, including respiratory tract infections.^3 4 4–8^ In particular, recent evidence suggests that short-term exposure to air pollution may increase the risk of worse outcomes in patients with COVID-19.^9^

Recent reports based on epidemiological time-series studies have found positive associations of short-term exposure to ambient air pollution and daily new confirmed cases of COVID-19.^10 11^ In fact, exposure to air pollution may impair airway immunity which may increase susceptibility to respiratory pathogens. Air pollution may also alter immune response to the infection and therefore lead to more severe disease.^5^ While recent reports indicate a possible link between short-term variations in outdoor concentrations of ambient air pollution and COVID-19, none of these studies have used individual-level data. Individual-level assessment would also allow more robust investigation of effect modification by individual characteristics (eg, age, sex), including those related to characteristics of COVID-19 diagnosis.

The general objective of this study was to test the hypothesis that short-term variations in outdoor concentrations of ambient air pollution increase the risk of emergency department (ED) visits for COVID-19. We also hypothesised that short-term variations in outdoor concentrations of ambient air pollution are associated with presence of pneumonia at the time of COVID-19 diagnosis in the ED. This is particularly important, because it may indicate a more severe manifestation of COVID-19.^12 13^

## Methods

### Study design

A time-stratified case-crossover study was conducted across 40 health regions in Canada in order to estimate associations between short-term variations in outdoor concentrations of ambient air pollution and risk of ED visits for COVID-19. The case-crossover design is an adaptation of the case-control study in which cases serve as their own control, and it is well suited for studying transient risk factors^14^ (online supplemental file 1 for additional details). The case’s exposure at the index time (ie, day of admission for COVID-19) is compared with its exposure at control time intervals, which are chosen using a time-stratified design.^15^ The index period is measured before the event and the control period is measured before and after the event.^16–18^ Referent intervals were selected from the same day of the week during the same month as the case interval (ie, 1:3 or 1:4 matching). For example, if the COVID-19 ED visit occurred on the second Wednesday in the month of January 2021, then the referent periods will be the other Wednesdays in January 2021, regardless if these occurred before or after the event. In fact, the time-stratified approach matches the exposure by day of the week and month to control for the influence of day-of-week effects. In addition, the time stratified approach is not subject to bias resulting from time trends and inherently takes into account seasonal trends in either the exposure levels and outcome data as well as for unmeasured subject-level risk factors (eg, obesity) that do no vary over short periods of time.^15 19 20^

10.1136/thoraxjnl-2021-217602.supp1 Supplementary data

### Health outcomes and data

All ED cases of COVID-19 occuring between 1 March 2020 and 31 March 2021 in 40 health regions across the provinces of Ontario (35 health regions) and Alberta (5 health regions) in Canada were identified from the National Ambulatory Care Reporting System (NACRS) database maintained by CIHI.^21^ Data were not available for other provinces for the time period under study given that only hospitals in Ontario and Alberta had mandated reporting for ED visits with diagnosis codes and were available when this study was initiated. Canada provides universal healthcare coverage for its residents; therefore, the NACRS database contain the large majority of ED visits in the provinces of Ontario and Alberta. Although the first patient being diagnosed with COVID-19 in Canada was identified on 25 January 2020, we limited our time period when the first wave of COVID-19 began around March 2020. Therefore, our study period overlapped the first wave as well as the second wave of COVID-19 cases, which occurred between October 2020 and February 2021. The following codes were identified from the main diagnosis to identify ED visits for COVID-19: International Classification of Diseases (ICD)-10th revision, Code U07.1 ‘COVID-19 case with virus identified by laboratory results’, and U07.2 ‘COVID-19 case diagnosed clinically or epidemiologically but laboratory results are inconclusive, not available or testing is not performed’. After identifying the COVID-19-cases, we searched secondary diagnostic fields for viral pneumonia (ICD-10 Code J12) and ‘pneumonia, unspecified’ (ICD-10 Code J18).^13^ We also extracted information on whether patients were admitted to the hospital after the ED visit, as an indicator of the severity of the disease. Demographic information, including age and sex, and postal code of residence were also extracted from the database. Cases without information on home address were excluded *a priori* to reduce exposure measurement error. We also excluded cases whose residential address was not within Ontario or Alberta, even if they were diagnosed there.

### Ambient air pollution and time-varying covariates

We extracted daily average concentrations of ambient fine particulate matter with diameters less than 2.5 µm (PM_2.5_), nitrogen dioxide (NO_2_) and ozone (O_3_) based on 10 km × 10 km grid surfaces from the Regional Air Quality Deterministic Prediction System operated by Environment and Climate Change Canada (ECCC) (https://weather.gc.ca/aqfm/index_e.html). Case and control periods (described below) with residential addresses within each grid of the surfaces were assigned exposures accordingly. Data for daily mean temperature and relative humidity were also provided by ECCC, which averaged the hourly mean temperature and relative humidity across 2.5 km × 2.5 km grid surfaces. We also extracted daily changes in mobility by health region which measures the change in average duration of time spent going to workplaces compared with the median for the same weekday in a prepandemic period (ie, 3 January 2020–6 February 2020). This was generated based on aggregated data of average mobility to workplaces from Google Account users who opted-in to location history for their account.^22^ In addition, we included daily information for each health region on the level of government interventions from the Oxford COVID-19 Government Response Tracker (OxCGRT) Government Response Index (on a scale of 1–100).^23^ Mobility data and OxCGRT were used as a proxy of traffic vehicle mobility and social distancing measures, which are likely associated both with the exposure (ie, ambient air pollution)^24^ and the outcome (COVID-19 ED visits).^25 26^ Finally, we estimated transmission dynamics of the virus and case ascertainment by including the daily effective reproduction number (R_t_) for each health region, which was used as a proxy for social contact mixing.

### Area-level predictors

We captured a number of predictors at the health region level in order to incorporate these in a meta-regression in order to reduce hererogeneity across regions. We first extracted from census data information on population density, percentage of the population with income less than the low income cut-off (LICO) in Canada, percentage of the population self-identified as Black and percentage of the health region population considered as urban.^27^ We also obtained the percentage of the population who rate their health as fair or poor. This information was obtained from the 2017 and 2018 Canadian Community Health Survey (CCHS).^28^ The CCHS is an annual national cross-sectional survey of individuals 12 years of age and over.

### Statistical analysis

We conducted a two-stage analysis. First, we used conditional logistic regressions with distributed lag non-linear models (DLNM) to examine associations between daily variations in ambient air pollution and COVID-19 ED visits for each of the 40 health regions. Second, we used a multivariate meta-regression model to pool the health region-specific estimates across all regions. We used R software (V.4.0.3; R DevelopmentCoreTeam) with the packages survival and dlnm for the time-stratified case-crossover analysis and mixmeta for the meta-regression. We used the package EpiNow2 to calculate R_t_.

### First-stage modelling

**Figure 1:**
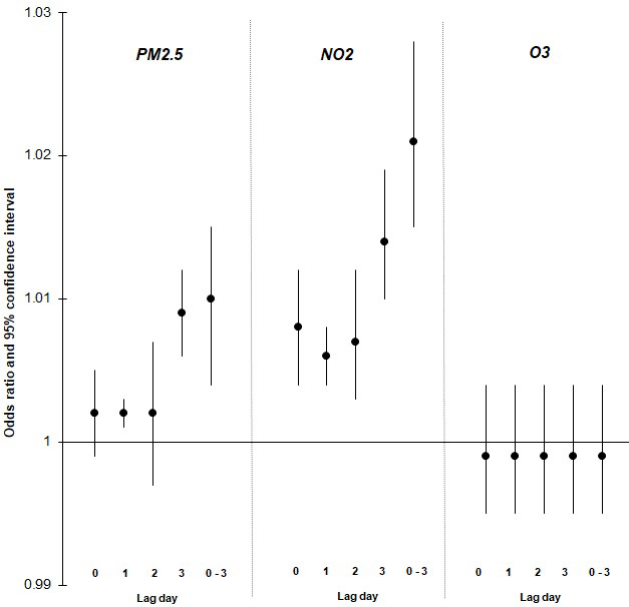
ORs and 95% CIs for associations between acute exposure to ambient air pollutants and emergency department visits for COVID-19. ORs reflect a 6.2 µg/m^3^ change in PM_2.5_, a 7.7 ppb change in NO_2_ and a 10.8 ppb change in O_3_. Models represent pooled health region-specific estimates derived using two-stage random effects meta-analysis and meta-regression. Models adjusted for daily mean ambient temperature, relative humidity, the effective reproduction number, the OxCGRT Government Response Index and population density and percentage of the population self-identified as Black as meta-predictors. NO_2_, nitrogen dioxide; O_3_, ozone; OxCGRT, Oxford COVID-19 Government Response Tracker; PM_2.5_, particulate matter with diameter less than 2.5 μm.

We ﬁt conditional logistic regressions in order to implement the time-stratiﬁed case-crossover analysis.^14^ We first estimated ORs along with their accompanying 95% CIs for the associations between IQRs of the distribution of differences between case and control periods of ambient air pollution over specific periods of exposure and COVID-19 ED visits for each health region. We first fitted models considering a lag period of up to 3 days prior to the index time or referent interval (ie, lag 0–3, where lag 0 is the exposure on the index time or referent interval and lag 1–3 are the three previous days), including single and cumulative effect of over several days. This lag period was identified based on prior studies on short term variations in outdoor concentrations of air pollution and health effects.^29 30^ In addition, our initial analysis showed that the association (see figure 1) was limited to the first few days following the admission. We made use of DLNMs in all models in order to fit exposure–response and the additional lag–response associations for each daily changing variable.^31^ Specifically, we modelled ambient air pollution using a linear function and in order to account for the lag response over 0–3 lagged days we incoporated an uncontrained lag function.^10 11^ We tested all models for adjustments for daily ambient temperature, daily relative humidity, average workplace mobility changes, OxCGRT Government Response Index and R_t_. In order to evaluate confounding by these daily varying covariates, we added covariates in an hierarchial manner in each model for each health region and compared the summed model fit from each model based on the Akaike information criteria (AIC). The lag response functions for the weather covariates were the same as the ambient air pollution variables. However, we modelled the lag function for the average workplace mobility changes, OxCGRT Government Response Index and R_t_ using a 13 lag period. We specified the same covariates consistently in the different health regions as well as with different distributed lags. Non-linearity of the exposure–response function for daily ambient temperature was accounted for using a quadratic B-spline with three internal knots placed at the 10th, 75th and 90th percentiles of location-specific temperature distributions. Similarly, non-linearity of the daily relative humidity exposure-response function was accounted for using natural cubic splines with three df.^32^ Model selection for exposure-response and lag-response functions was based on the AIC as well as visual inspections of preliminary findings. We finally made use of directed acyclic graph (DAG) in order to identify the final potential confounding variables.

### Second-stage modelling

Multivariate meta-regression analysis was used to pool the health region-specific effect estimates.^33^ This meta-analysis approach allowed the derivation of improved estimates of air pollution-COVID-19 associations at the health region level, defined as best linear unbiased predictions. Best linear unbiased predictions borrow information across units within the same hierarchical level (ie, individual and health region level) and can provide more accurate estimates, especially in locations with small number of cases of COVID-19. We fit the meta-regression with and without health-region meta-predictors in order to estimate the overall associations and evaluate how the between-health region variability of the air pollution–COVID-19 association are explained by the meta-predictors. We used a multivariate Wald test for the significance of the meta-predictors and Cochran’s Q-test and I^2^ statistic to examine how the residual heterogeneity changes with different meta-predictors.

### Subgroup analysis

We conducted stratified analyses within the first-stage and second-stage modelling domains described above in order to obtain ORs by predefined categories of age (0–64, ≥65), sex, level of certainty of the COVID-19 diagnosis (confirmed/suspected), whether cases were hospitalised or not, and whether the ED visit was also coded as viral pneumonia. We evaluated whether those characteristics could modify associations between daily variations in ambient air pollution and COVID-19 ED visits. To test the statistical significance of differences between the estimates of the strata, we used a multilevel meta-regression model where we considered the individual-level characteristics from the same healh region as multivariate outcomes sharing the same health region random effect. We subsequently used a Wald test to evaluate significance of effect modification.^33^ We also conducted an analysis of the impact of air pollution on a comparator outcome, using the same time period and health regions we used for the COVID-19 analysis. We, therefore, extracted cases of myocardial infarction (MI) (ICD-10: I21) and assesed the associations between ambient air pollution and MI ED visits. We also assessed whether impacts of air pollution on COVID-19 ED visits were different by whether patients came from institutional settings (eg, long term healthcare facility, correctional facility) and non-institutional settings. This was investigated given that exposure to air pollution for those in institutions may be different from the rest of the population given they are more confined in the indoor environment. We also assessed the impacts of air pollution on COVID-19 by stratifying for the time period of the study (ie, March 2020 to September 2020 vs October 2020 to March 2021), in order to investigate whether different dynamics may have been present in the period following the first wave of the pandemic versus the second wave of the pandemic in Canada.

## Results

In total, there were 78 255 ED visits for COVID-19 in Alberta and Ontario between 1 March 2020 and 31 March 2021. Most cases were observed in Ontario, were less than 65 years of age (75.5%) and were laboratory confirmed (84.4%). Characteristics of the study population are shown in table 1. Mean daily concentrations of air pollutants were 6.48 µg/m^3^ for PM_2.5_, 7.96 ppb for NO_2_ and 24.60 ppb for O_3_. The average daily mean temperature was 2.81°C, varying from −33.34°C to 28.51°C (IQR of 10.75°C). Table 2 shows the descriptive statistics of weather and air quality parameters in Alberta and Ontario during the study period. We also present (online supplemental table S1) the distribution of the IQRs across months of the study for each pollutant. Online supplemental table S2 shows Pearson correlation coefficients among the air pollutants and other daily varying variables. PM_2.5_ was highly correlated with NO_2_ (*r*=0.87), but weakly correlated with O_3_ (*r*=−0.11).

**Table 1:** Number of emergency department visits of COVID-19 cases across 40 health regions between 1 March 2020 and 31 March 2021 in Canada by specific characteristics

Variable	No of ED visits (%)
Age (in years)	
<65	59 074 (75.5)
≥65	19 181 (24.5)
Sex	
Male	39 774 (50.8)
Female	38 469 (49.2)
COVID-19 case status	
Confirmed	66 046 (84.4)
Suspected	12 209 (15.6)
Pneumonia	
Yes	12 951 (16.6)
No	65 304 (83.5)
Hospitalised	
Yes	18 299 (24.3)
No	59 256 (75.7)
Province	
Alberta	12 753 (12.3)
Ontario	65 505 (83.7)
Total visits	78 255

ED, emergency department.

**Table 2:** Daily concentrations of ambient air pollutants and daily-varying covariates

Variable	Mean (SD)	Median	IQR	Range
PM_2.5_ (µg/m^3^)	6.48 (5.28)	5.21	6.24	0.02–42.43
NO_2_ (ppb)	7.96 (5.78)	6.70	7.69	0.01–31.30
O_3_ (ppb)	24.60 (7.94)	24.48	10.75	3.33–66.18
Temperature (^o^C)	2.81 (9.07)	1.36	10.75	−33.34–28.51
Relative humidity (%)	73.51 (12.53)	74.68	17.33	30.81–99.41
Workplaces mobility change* (%)	−45.1	−51.0	30	−89–20
Effective reproduction no	1.04 (0.17)	1.00	0.20	0.60–1.90
OxCGRT Government Response Index	63.14 (12.91)	67.0	11.0	3.0–81.0

*Daily workplaces mobility per cent change from baseline (vs 3 January 2020 to 6 February 2020).

NO2, nitrogen dioxide; O3, ozone; OxCGRT, Oxford COVID-19 Government Response Tracker; PM2.5, particulate matter with diameter less than 2.5 μm.

Figure 1 shows the associations between the ambient air pollutants (PM_2.5_, NO_2_ and O_3_) and ED visits for COVID-19. Statistically significant associations were observed between PM_2.5_ and COVID-19 ED visits for 3 days lagged exposure (ie, lag 3) (OR 1.009; 95% CI 1.006 to 1.012, per 6.2 µg/m^3^) and the cumulative effects over lags 0–3 days (OR 1.010; 95% CI 1.004 to 1.015, per 6.2 µg/m^3^). In models investigating NO_2_, we also found an association over lags 0–3 days (OR 1.021; 95% CI 1.015 to 1.028, per 7.7 ppb). We did not find indication of an association for exposure to O_3_ and COVID-19 ED visits (results also shown in online supplemental table S2). All models were adjusted for daily variations in temperature, humidity, R_t_ as well as the OxCGRT Government Response Index (as per the DAG in online supplemental figure S2). We found that adding workplaces mobility did not improve the model fit and that the OxCGRT Government Response Index was better in terms of improving model fit We also found that accounting for meta-predictors in meta-regression models (ie, population density and percentage of the population self-identified as Black) further reduced the between-location heterogeneity, but the between health region heterogeneity remained statistically significant (results shown in online supplemental tables S4–S6)

The results of the stratified analyses examining the relationship between the cumulative effects of air pollutants over 0–3 days and ED visits for COVID-19 across individual-level characteristics are shown in table 3. When stratifying analyses by hospitalisation status, we found that the effect of PM_2.5_ on ED visits for COVID-19 was higher among those hospitalised (OR 1.023; 95% CI 1.015 to 1.031 per 6.2 µg/m^3^) compared with those not hospitalised following their ED visit (OR 0.992; 95% CI 0.980 to 1.004, per 6.2 µg/m^3^) (p value for effect modification=0.04). We also found higher effect estimates for the impact of O_3_ on ED visits for COVID-19 among those hospitalised (p value for effect modification=0.01). There was no conclusive evidence of effect modification by the other investigated characteristics.

**Table 3:** ORs* and 95% CIs for associations between the cumulative effects of ambient air pollutants over 0–3 days (per IQR increase) and emergency department visits for COVID-19

Characteristics	PM_2.5_	NO_2_	O_3_
Age (in years)			
<65	1.016 (1.007–1.026) I^2^=50.6% (<0.01)	1.024 (1.016–1.033) I^2^=21.9% (0.15)	1.002 (0.996–1.009) I^2^=16.1% (0.22)
≥65	1.012 (1.001–1.023) I^2^=25.9% (0.11)	1.034 (1.018–1.050) I^2^=46.7% (<0.01)	0.994 (0.989–1.000) I^2^=23.8% (0.13)
P value for effect modification	0.72 I^2^=43.4% (<0.01)	0.99 I^2^=44.6% (<0.01)	0.52 I^2^=52.6% (<0.01)
Sex			
Male	1.013 (1.003–1.022) I^2^=37.7% (0.03)	1.037 (1.022–1.053) I^2^=50.4% (0.01)	1.001 (0.994–1.009) I^2^=56.9% (<0.01)
Female	1.017 (1.008–1.026) I^2^=41.5% (0.01)	1.014 (1.006–1.023) I^2^=87.1% (<0.01)	1.003 (0.996–1.009) I^2^=54.2% (<0.01)
P value for effect modification	0.49 I^2^=41.4% (<0.01)	0.34 I^2^=41.4% (<0.01)	0.51 I^2^=41.4% (<0.01)
COVID-19 case status			
Confirmed	1.012 (1.004–1.021) I^2^=47.2% (<0.01)	1.028 (1.021–1.036) 29.1% (0.08)	1.000 (0.994–1.006) 70.5% (<0.01)
Suspected	1.010 (1.000–1.021) I^2^=0.0% (0.68)	0.985 (0.969–1.000) 0.0% (0.52)	1.009 (1.005–1.013) 30.4% (0.07)
P value for effect modification	0.84 I^2^=41.9% (<0.01)	0.27 I^2^=30.6% (0.02)	0.31 I^2^=60.3% (<0.01)
Pneumonia status			
Yes	0.995 (0.978–1.013) I^2^=43.8% (<0.01)	1.017 (1.001–1.033) I^2^=33.8% (0.05)	0.998 (0.991–1.004) I^2^=20.4% (0.18)
No	1.015 (1.011–1.019) I^2^=32.1% (0.06)	1.029 (1.021–1.037) I^2^=34.1% (0.04)	1.001 (0.995–1.008) I^2^=65.2% (<0.01)
P value for effect modification	0.64 I^2^=43.1% (<0.01)	0.84 I^2^=37.3% (0.01)	0.41 I^2^=50.0% (<0.01)
Hospital			
Yes	1.023 (1.015–1.031) I^2^=44.2% (0.01)	1.020 (1.008–1.032) I^2^=41.5% (0.02)	1.005 (0.998–1.011) I^2^=64.1% (<0.01)
No	0.992 (0.980–1.004) I^2^=37.5% (0.03)	1.027 (1.020–1.034) I^2^=21.3% (0.16)	0.993 (0.988–0.998) I^2^=34.7% (0.04)
P value for effect modification	0.04 I^2^=47.7% (<0.01)	0.84 I^2^=41.4% (0.00)	0.01 I^2^=52.7% (<0.01)

I^2^: The variance due to heterogeneity estimated by the I²-statistic for the strata models and the models when calculating the p value for effect modification. In parentheses, the p values for the statistical significance of heterogeneity are reported.

Models represent pooled health region-specific estimates derived using two-stage random effects meta-analysis and meta-regression.

ORs reflect a 6.2 µg/m^3^ change in PM2.5, a 7.7 ppb change in NO_2_ and a 10.8 ppb change in O_3_

*Models represent pooled health region-specific estimates derived using two-stage random effects meta-analysis and meta-regression. ORs reflect a 6.2 µg/m^3^ change in PM_2.5_, a 7.7 ppb change in NO_2_ and a 10.8 ppb change in O_3_. Models adjusted for daily mean ambient temperature, relative humidity, the effective reproduction number, the OxCGRT Government Response Index and population density and percentage of the population self-identified as Black as meta-predictors.

NO2, nitrogen dioxide; O3, ozone; OxCGRT, Oxford COVID-19 Government Response Tracker; PM2.5, particulate matter with diameter less than 2.5 μm.

In sensitivity analyses, we did not find effect modification (p values for effect modification >0.58) when stratified by whether patients came from institutional settings (eg, long term healthcare facility, correctional facility) and non-institutional settings (online supplemental table S7). However, we found higher impacts of exposure to NO_2_ on COVID-19 ED visits during the time period from October 2020 to March 2021 (OR 1.034; 95% CI 1.026 to 1.042, per 7.7 ppb) compared with the period from March 2020 to September 2020 (OR 1.001; 95% CI 0.978 to 1.023, per 7.7 ppb) (p value for effect modification=0.03). We found the opposite to be true for O_3,_ where the impacts on COVID-19 ED visits were positive during the time period from March 2020 to September 2020 (OR 1.035; 95% CI 1.025 to 1.046, per 10.8 ppb) and were not statistically significant during the time period from October 2020 to March 2021 (OR 0.994; 95% CI 0.988 to 1.000, per 10.8 ppb). We also found that removing Rt from the models as an adjustment variable, while keeping all other variables and meta-predictors in the models, slightly increased the point estimates for most models (eg, OR 1.015; 95% CI 1.007 to 1.023, per 6.2 µg/m^3^ for the cumulative effect over 0 to 3 days for PM_2.5_ & OR 1.042; 95% CI 1.035 to 1.050, per 7.7 ppb for NO_2_). In addition, when removing the OxCGRT Government Response Index from the models, a non statistically significant OR was observed for PM_2.5_ (OR 0.997; 95% CI 0.987 to 1.007, per 6.2 µg/m^3^) while a higher effect estimate was observed for NO_2_ (OR 1.035; 95% CI 1.029 to 1.042, per 7.7 ppb for NO_2_). Finally, when evaluating a comparator outcome, we found an association between the cumulative effect of PM_2.5_ over 3 days and the risk of MI ED visits (OR 1.003; 95% CI 1.001 to 1.006, per 1.8 µg/m^3^).

## Discussion

This case-crossover study using individual-level data showed overall positive associations between short term changes in outdoor PM_2.5_ and NO_2_ and ED visits for COVID-19. We also found that the associations between PM_2.5_ and COVID-19 were stronger in patients hospitalised, than in COVID-19 patients attending the ED that were not hospitalised. We also found a positive finding for O_3,_ only among those hospitalised.

A small number of studies to date have applied time-series designs to investigate acute air pollution impacts on COVID-19 case counts. These studies have relied on aggregated administrative data (eg, city, county or region). A study conducted in 120 cities in China found associations between exposure to PM_2.5_, PM_10_, NO_2_ and O_3_ in the last 2 weeks and daily COVID-19 confirmed cases.^34^ In another Chinese study focusing on 63 cities, authors found positive associations between exposure to PM_10_ and PM_2.5_ over a lag period of 7–14 days and daily counts of COVID-19 cases.^10^ A third study conducted in China further identified daily interactions between air pollution and meteorological factors and COVID-19 confirmed cases.^35^ In addition, a study conducted in a single New York area in the USA did not observe an effect of PM_2.5_ on COVID-19 confirmed cases, but some evidence of an effect for O_3_. A study conducted in the Lombardy region in Italy found that short term variations in PM_10_, PM_2.5_ and O_3_ concentrations, especially at short lags, were associated with increased COVID-19 incidence.^36^ Finally, a study conducted in Ontario, Canada from January to June 2020 found a suggestive positive association between 1 week averaged O_3_ exposure and COVID-19 incidence among institutional outbreak cases (eg, long-term care home, hospital, correctional facility).^37^ Those studies have raised concerns of potential impacts of air pollution exposure on COVID-19 incidence. However, some of these studies have limitations due to lack of adjustment for government response interventions, changes in population mobility and failure to adjust aggregated data for important confounders.^38^ Another limiting factor is that these studies relied on relatively short periods of no more than a few months, thereby limiting contrasts in exposures and outcome frequency, and the ability to capture multiple seasons. Furthermore, while a number of studies have found associations between chronic exposure to air pollution and COVID-19 transmission rates using mainly ecological designs,^9^ some concerns have arisen about methodological issues.^39 40^

A potential mechanism linking short term changes in ambient air pollution and COVID-19 ED visits might be related to the effects of the pollutants on the immune response, influencing the severity of disease among those already infected. Previous evidence has shown that exposure to PM_2.5_ 41 42 and gaseous pollutants^43 44^ can lead to immune system dysregulation such as overexpression of inflammatory cytokines and chemokines.^44 45^ Immune system dysregulation can lead to inappropriate local and systemic immune responses and the subesquent rapid spread of the virus, leading to severe COVID-19 disease and Acute Respiratory Distress Syndrome. In addition, dysfunctional immune system responses could lead to a lowered threshold for acquiring a new pathogenic or opportunistic infection, exacerbating the existing infection, or exacerbation of certain existing non-infection diseases such as asthma.^46^ Any of the above could be detrimental in COVID-19 patients. Thus, the hypothesis that air pollutants may increase the risk of COVID-19 infection and potentially lead to a more severe disease is biologically plausible. The fact that we found positive associations between PM_2.5_, NO_2_ and O_3_ COVID-19 in the relatively low pollutant environments of Alberta and Ontario, and the fact that some of the associations were stronger in those hospitalised, are consistent with this hypothesis.

We also found that the proportion of between-health region variability, obtained through the heterogeneity statistics, decreased when adding the population density and the proportion of of Black residents as meta-predictors. In fact, there have been numerous reports of increased morbidity and mortality from COVID-19 in Black populations or with a higher concentrations of minorities.^47–50^ Other reports have also shown that areas with a higher population density was associated with increased incidence of case counts and mortality for COVID-19.^51 52^ These factors may be indicative of increased vulnerability and/or increased social contacts, which may be important to account for in future epidemiological studies assessing the risk of air pollution on COVID-19.

This study has several strengths. The case-crossover study design is well suited for studying the effects of transient risk factors.^14^ We performed individual-level analysis of the associations between daily levels of several air pollutants and COVID-19. We accounted for changes in health region-level mobility and social distancing and we also included daily variations in the effective reproduction number (R_t_) in the models. We took advantage of the home addresses of the cases, and the geospatial resolution of our exposure assessment is a major improvement to the city-level time-series more commonly used in epidemiological studies.

This study has several limitations. First, we restricted our case selection to those receiving medical assistance at the ED. Consequently, our findings cannot be generalised to cases not seeking care through the ED. We also observed a higher number of cases in those aged less than 65 years, which may be counter intuitive to reports showing higher number of cases in those over 65, in particular during the first wave of the pandemic.^53^ However, we did not calculate the incidence rates of ED visits for COVID-19 by age group, which was beyond the scope of our study, but we believe rates were likely higher in those aged over 65. Second, we used concurrent diagnosis of pneumonia as a proxy for the severity of COVID-19 symptoms. While this may be justified on the basis that most severe forms of COVID-19 commonly lead to pulmonary manifestations,^12^ we did not per se have any direct measure of severity or a validated severity index at our disposal. In addition, EDs can be busy at times, and some cases may have had concurrent pneumonia without the appropriate diagnosis finding its way the patient records. Also, in our data, we cannot clearly distinguish whether pneumonia resulted from COVID-19 infection or the opposite. In the data we extracted, COVID-19 was the main reason for visiting the hospital and pneumonia was reported as a secondary diagnosis. In fact, in our data, we did not extract any admission where pneumonia was the primary diagnosis and COVID-19 as a secondary diagnosis. However, it is unlikely that the reporting of coexisting pneumonia would have been the result of systematic diagnostic misclassification. We also used the hospitalisation status as a measure of COVID-19 severity, but we did not have the actual length of stay in the hospital, which may be an important endpoint to consider. In addition, it is unclear whether being hospitalised reflects a measure of COVID-19 severity or may be related to other factors (eg, admissions practices or general rate of admissions from ED attendance). Our study may be limited by unmeasured and residual confounding. This may include confounding caused by individual behaviours that were unmeasured and which may be correlated with both air pollution and risk of COVID-19 ED visits. For instance, individual-level mask-wearing may have reduced personal air pollution exposure as well as virus transmission. In Canada, towards the end of May 2020, officials recommended Canadians to wear non-medical face masks when maintaining a two-metre distance was not possible. Although the specific recommendations were implemented using different approaches across provinces, we believe it is unlikely that this would confound strongly our findings. It is unlikely that mask wearing behaviours were substantially different between case and control periods. To account for this, we adjusted for area-level government responses and the effective reproduction number. In addition, the case-crossover design explicitly controls for subject-specific factors that do not appreciably vary over the short term. Some exposure misclassification is possible if the cases did not spend time at the vicinity of their homes before the ED visit. This error would be most likely for NO_2_ exposures, as within-city spatial variations are greater for NO_2_ than for O_3_ or PM_2.5_, and exposure models may not adequately represent spatial differences in NO_2_ exposures over large geographic areas. However, the study was conducted at the time of COVID-19 pandemic, and the residents may have stayed at their homes more than usual. In addition, air pollution monitoring occurred outdoors, which does not account for differences of indoor air pollution exposure. However, the inherent nature of the case-crossover study compares case periods to control periods occurring during the same month and day-of-the-week as the case periods, which controls for non-time dependent confounders. Previous studies have shown that this type of measurement error (ie, lack of indoor air pollution exposure) results in non-differential exposure misclassification, which likely underestimates the risks of air pollution.^18 53^

Finally, we did not have individual level data on race and socioeconomic status (SES), since there is evidence that racialised and lower SES populations are at greater risk. Without these data, modification of the effect of air pollution by these factors could not be examined.

In summary, this individual-level case-crossover study provided evidence of an increased risk of ED visit for COVID-19 associated with short-term exposure to PM_2.5_ and NO_2_ and, to a lesser extent, O_3_. Consistent with our hypothesis, the association was stronger for cases with an hospitalisation, which may indicate a more severe disease manifestation. Additional research regarding the relationships between acute exposure to ambient air pollution and COVID-19 is needed.

## Data Availability

Data may be obtained from a third party and are not publicly available. Data are on secured server and cannot be accessible to the public.
