# Supplementary material for: Short-term exposure to ambient air pollution and individual emergency department visits for COVID-19: a case-crossover study in Canada
Source: Thorax. 2022 Mar 30:thoraxjnl-2021-217602. doi: 10.1136/thoraxjnl-2021-217602 (PMC8983401; doi:10.1136/thoraxjnl-2021-217602)

BMJ Publishing Group Limited (BMJ) disclaims all liability and responsibility arising from any reliance placed on this supplemental material which has been supplied by the author(s)

Supplementary material

Short term exposure to ambient air pollution and individual emergency department visits for COVID-19: a case-crossover study in Canada

Table S1. Daily interquartile ranges of ambient air pollutants across months of the study

Month	$PM_{2.5}$ (μg/m^3)	NO_2 (ppb)	O_3 (ppb)
March	8.80	8.34	9.19
April	5.80	9.88	10.11
May	5.16	7.22	8.16
June	5.63	8.68	7.43
July	7.20	8.92	10.88
August	6.00	8.12	15.14
September	6.57	9.59	12.62
October	6.06	6.69	12.44
November	4.48	5.69	8.94
December	7.26	6.78	9.93
January	4.97	7.05	10.15

Table S2. Descriptive statistics and Pearson's correlation coefficients between different daily-changing variables.

Variables	Variables							
	$PM_{2.5}$	NO_2	O_3	Temperature	Relative Humidity	Workplaces mobility change	Effective reproduction number	OxCGRT Government Response Index
$PM_{2.5}$	1.00							
NO_2	0.87	1.00						
O_3	-0.11	-0.32	1.00					
Temperature	0.26	0.18	-0.07	1.00				
Relative Humidity	0.14	0.04	0.09	0.09	1.00			
Workplaces mobility change	-0.10	-0.15	-0.04	0.11	0.13	1.00		
Effective reproduction number	0.05	0.11	0.15	-0.42	0.24	0.43	1.00	
OxCGRT Government Response Index	-0.04	-0.06	0.02	0.00	-0.16	-0.71	-0.70	1.00

Table S3. Odds ratios[1] (ORs) and 95% CIs for associations between acute exposure to ambient air pollutants and emergency department visits for COVID-19. ORs reflect a 6.2 μg/m^3 change in $PM_{2.5}$, a 7.7 ppb change in NO_2 and a 10.8 ppb change in O_3.

**Lag period**	**PM2.5**	**NO2**	**O3**
Lag 0	1.002 (0.990 – 1.005)	1.008 (1.004 – 1.012)	0.999 (0.995 – 1.004)
Lag 1	1.002 (1.001 – 1.003)	1.006 (1.004 – 1.008)	0.999 (0.995 – 1.004)
Lag 2	1.002 (0.997 – 1.007)	1.007 (1.003 – 1.012)	0.999 (0.995 – 1.004)
Lag 3	1.009 (1.006 – 1.012)	1.014 (1.010 – 1.019)	0.999 (0.995 – 1.004)
Cumulative 0 – 3	1.010 (1.004 – 1.015)	1.021 (1.015 – 1.028)	0.999 (0.995 – 1.004)
I^2 (P value for heterogeneity)[2]	42.8% (<0.01)	46.2% (<0.01)	62.9% (<0.01)

[1]Models represent pooled health region-specific estimates derived using two-stage random effects meta-analysis and meta-regression. Models adjusted for daily mean ambient temperature, relative humidity, the effective reproduction number, the OxCGRT Government Response Index and population density and percentage of the population self-identified as Black as meta-predictors.

[2]The variance due to heterogeneity estimated by the I^2-statistic.

Table S4. Second-stage random-effects meta-analysis and meta-regression models for the associations between $PM_{2.5}$ (per 6.2 μg/m^3) and COVID-19 ED visits: multivariate Wald test on significance of each meta-predictor in explaining variation in overall associations, Cochran Q test for heterogeneity and I^2 statistics for residual heterogeneity.

**Meta-predictors**	**AIC**	**Cochran Q**	**Q test (p value)**	I^2	**Effect modification by meta-predctor (p value)**
Base model	31.5	113.6	< 0.001	65.7%	-
< Low Income Cut-off (%)	24.8	95.3	< 0.001	60.1%	0.89
Population density	24.4	93.8	< 0.001	59.5%	0.77
Black (%)	28.3	109.8	< 0.001	65.4%	0.15
Poor health (%)	28.3	112.9	< 0.001	66.3%	0.08
Urban (%)	27.1	113.5	< 0.001	66.5%	0.22
Overweight or obese (%)	25.1	83.6	< 0.001	54.6%	0.85
Long term $PM_{2.5}$	25.1	83.6	< 0.001	54.6%	0.85
+ Population density<br>+ Black (%)	43.3	36.5	0.003	42.8%	0.16<br>0.05

Different meta-regression models are being presented: base model (i.e. only including pooled ORs) and models with different meta-predictors. Random-effects multivariate meta-regression models were used to test potential effect modification by between-city differences in meta-predictors. The outcome variables in the meta-regression models in this study were the pooled estimates and the explanatory variables (i.e. potential effect modifiers) were the continuous variables at the health region level. Effect modification was considered statistically significant if the effect modifier's p-value was less than 0.05.

Table S5. Ssecond-stage random-effects meta-analysis and meta-regression models for the associations between NO_2 (per 7.7 ppb) and COVID-19 ED visits: multivariate Wald test on significance of each meta-predictor in explaining variation in overall associations, Cochran Q test for heterogeneity and I^2 statistics for residual heterogeneity.

Meta-predictors	AIC	Cochran Q	Q test (p value)	I^2	Effect modification by meta-predctor (p value)
Base model	29.4	169.6	< 0.001	77.0%	-
< Low Income Cut-off (%)	35.3	116.0	< 0.001	67.3%	0.43
Population density	35.9	116.8	< 0.001	67.5%	0.45
Black (%)	35.3	166.0	< 0.001	77.1%	0.51
Poor health (%)	34.7	169.0	< 0.001	77.5%	0.53
Urban (%)	33.5	165.2	< 0.001	77.0%	0.35
Overweight or obese (%)	35.0	95.7	< 0.001	60.3%	0.55
Long term $PM_{2.5}$	34.7	169.0	< 0.001	77.5%	0.53
+ Population density<br>+ Black (%)	40.0	68.7	0.001	46.2%	0.08<br>0.08

Different meta-regression models are being presented: base model (i.e. only including pooled ORs) and models with different meta-predictors. Random-effects multivariate meta-regression models were used to test potential effect modification by between-city differences in meta-predictors. The outcome variables in the meta-regression models in this study were the pooled estimates and the explanatory variables (i.e. potential effect modifiers) were the continuous variables at the health region level. Effect modification was considered statistically significant if the effect modifier's p-value was less than 0.05.

Table S6. Ssecond-stage random-effects meta-analysis and meta-regression models for the associations between O_3 (per 10.8 ppb) and COVID-19 ED visits: multivariate Wald test on significance of each meta-predictor in explaining variation in overall associations, Cochran Q test for heterogeneity and I^2 statistics for residual heterogeneity.

Meta-predictors	AIC	Cochran Q	Q test (p value)	I^2	Effect modification by meta-predctor (p value)
Base model	100.7	115.4	< 0.001	66.2%	-
< Low Income Cut-off (%)	92.9	111.1	< 0.001	65.8%	0.93
Population density	92.8	110.7	< 0.001	65.7%	0.68
Black (%)	93.0	100.0	< 0.001	62.0%	0.98
Poor health (%)	92.0	113.7	< 0.001	66.6%	0.22
Urban (%)	95.6	114.0	< 0.001	66.9%	0.16
Overweight or obese (%)	93.3	107.7	< 0.001	64.7%	0.82
Long term $PM_{2.5}$	95.6	114.0	< 0.001	66.9%	0.16
+ Population density<br>+ Black (%)	85.2	100.0	< 0.001	62.9%	0.62<br>0.75

Different meta-regression models are being presented: base model (i.e. only including pooled ORs) and models with different meta-predictors. Random-effects multivariate meta-regression models were used to test potential effect modification by between-city differences in meta-predictors. The outcome variables in the meta-regression models in this study were the pooled estimates and the explanatory variables (i.e. potential effect modifiers) were the continuous variables at the health region level. Effect modification was considered statistically significant if the effect modifier's p-value was less than 0.05.

Table S7. Odds ratios[1] (ORs) and 95% CIs for associations between the cumulative effects of ambient air pollutants over 0 to 3 days (per interquartile range increase) and emergency department visits for COVID-19, stratified by whether patients came from institutional settings and by time period of the study. ORs reflect a 6.2 μg/m^3 change in $PM_{2.5}$, a 7.7 ppb change in NO_2 and a 10.8 ppb change in O_3. Models represent pooled health region-specific estimates derived using two-stage random effects meta-analysis and meta-regression.

Characteristics	$PM_{2.5}$	NO_2	O_3
Institutional setting			
Yes	1.012 (0.992 – 1.033)<br>I^2 = 8.7% (0.34)	1.009 (0.989 – 1.029)<br>I^2 = 0.0% (0.48)	1.008 (0.994 – 1.023)<br>I^2 = 41.1% (0.02)
No	1.014 (1.007 – 1.020)<br>I^2 = 40.4% (0.02)	1.027 (1.019 – 1.035)<br>I^2 = 30.7% (0.08)	1.000 (0.995 – 1.005)<br>I^2 = 63.1% (<0.01)
P value for effect modification	0.58	0.84	0.95
I^2 (P value for heterogeneity)[2]	I^2 = 29.1% (0.03)	I^2 = 23.5% (0.07)	I^2 = 53.5% (<0.01)
Time period			
March 2020 to September 2020	1.024 (1.017 – 1.030)<br>I^2 = 0.0% (0.68)	1.001 (0.978 – 1.023)<br>I^2 = 18.3% (0.20)	1.035 (1.024 – 1.046)<br>I^2 = 57.3% (<0.01)
October 2020 to March 2021	1.018 (1.013 – 1.023)<br>I^2 = 24.2% (0.13)	1.034 (1.026 – 1.042)<br>I^2 = 33.3% (0.05)	0.994 (0.988 – 1.000)<br>I^2 = 0.0% (0.50)
P value for effect modification	0.55	0.03	(<0.01)
I^2 (P value for heterogeneity)[2]	I^2 = 5.2% (0.37)	I^2 = 43,6% (<0.01)	I^2 = 71.3% (<0.01)

[1] Models represent pooled health region-specific estimates derived using two-stage random effects meta-analysis and meta-regression. ORs reflect a 6.2 μg/m^3 change in $PM_{2.5}$, a 7.7 ppb change in NO_2 and a 10.8 ppb change in O_3. Models adjusted for daily mean ambient temperature, relative humidity, the effective reproduction number, the OxCGRT Government Response Index and population density and percentage of the population self-identified as Black as meta-predictors.

I^2 : The variance due to heterogeneity estimated by the I^2-statistic for the strata models and the models when calculating the p value for effect modification. In parentheses, the p values for the statistical significance of heterogeneity are reported.

Table S8. Odds ratios[1] (ORs) and 95% CIs for associations between acute exposure to ambient air pollutants and emergency department visits for myocardial infarction. ORs reflect a 1.8 μg/m³ change in $PM_{2.5}$, a 2.3 ppb change in NO_2 and a 11.7 ppb change in O_3 (N = 26,437). The interquartile ranges were based on cases of MI during the time period of March 1st 2020 and March 31st 2021, in Alberta and Ontario.

Lag period	PM2.5	NO2	O3
Lag 0	1.004 (0.984 – 1.025)	1.007 (0.979 – 1.035)	0.970 (0.915 – 1.028)
Lag 1	1.007 (0.987 – 1.028)	1.001 (0.974 – 1.029)	0.991 (0.936 – 1.049)
Lag 2	1.014 (0.993 – 1.035)	0.995 (0.959 – 1.033)	0.996 (0.929 – 1.068)
Lag 3	0.999 (0.978 – 1.020)	0.988 (0.953 – 1.024)	0.985 (0.915 – 1.060)
Cumulative 0 – 3	1.003 (1.001 – 1.006)	0.998 (0.995 – 1.000)	0.998 (0.997 – 0.998)
I^2 (P value for heterogeneity)[2]	2.5% (0.95)	0.0% (0.99)	0.0% (0.98)

[1]Models represent pooled health region-specific estimates derived using two-stage random effects meta-analysis and meta-regression. Models adjusted for daily mean ambient temperature, relative humidity and the OxCGRT Government Response Index

[2]The variance due to heterogeneity estimated by the I^2-statistic.

Figure S1. Odds ratios[1] (ORs) and 95% CIs for associations between PM2.5 (per 6.2 μg/m^3) and emergency department visits for COVID-19 for lags 0 to 21 days. Models represent pooled health region-specific estimates derived using two-stage random effects meta-analysis and meta-regression. Models adjusted for daily mean ambient temperature, relative humidity, the effective reproduction number, the OxCGRT Government Response Index and population density and percentage of the population self-identified as Black as meta-predictors.

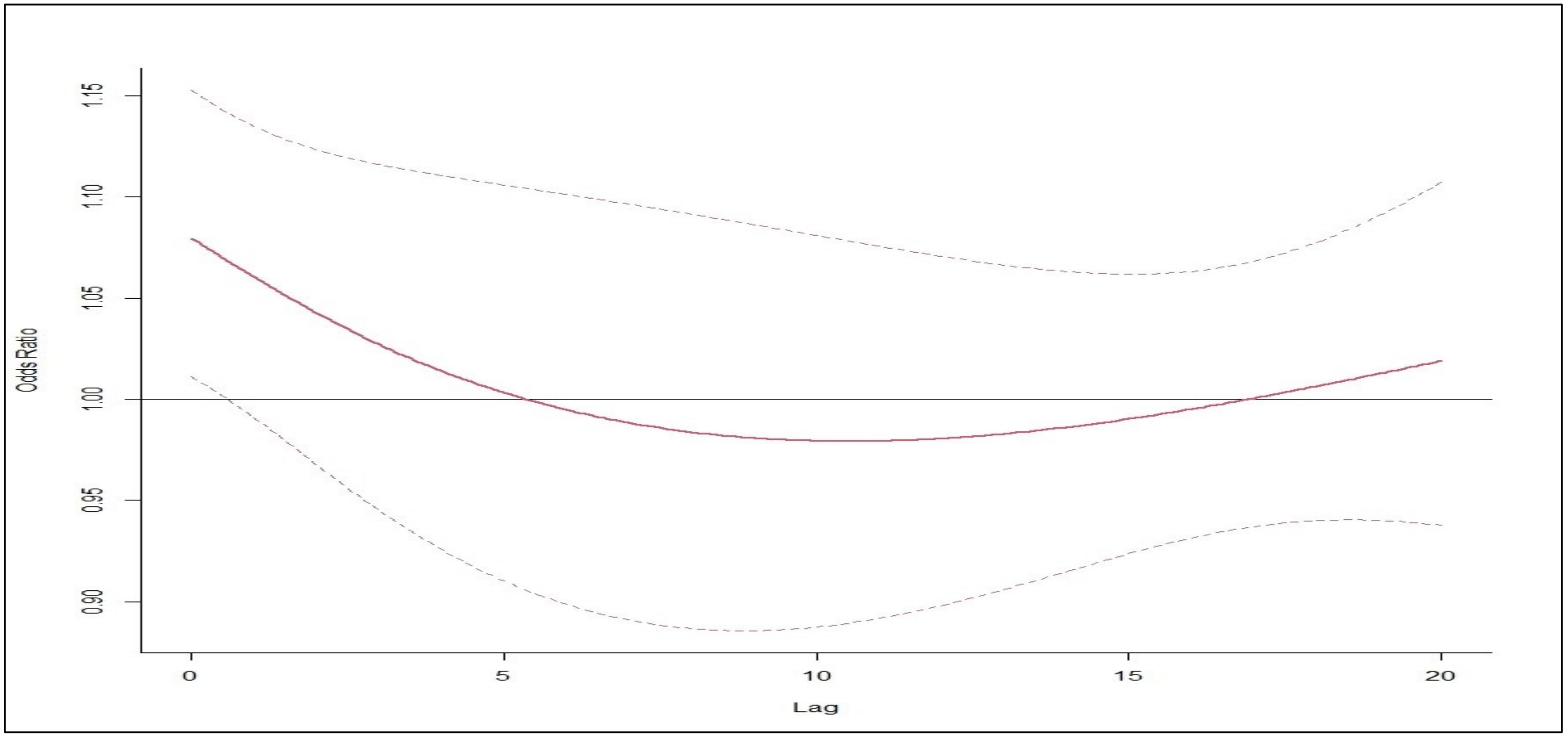

BMJ Publishing Group Limited (BMJ) disclaims all liability and responsibility arising from any reliance placed on this supplemental material which has been supplied by the author(s)

Figure S2. Directed acyclic graph for estimating the direct effect of ambient air pollution exposure on COVID-19 ED visits. **Parameters in red are potential confounding factors**. Green line: causal path. According to the DAG, the minimal sufficient adjustment for estimating the total effect of ambient air pollution on COVID-19 ED visits is: ambient temperature, Government Stringency Index, Relative humidity, Rt

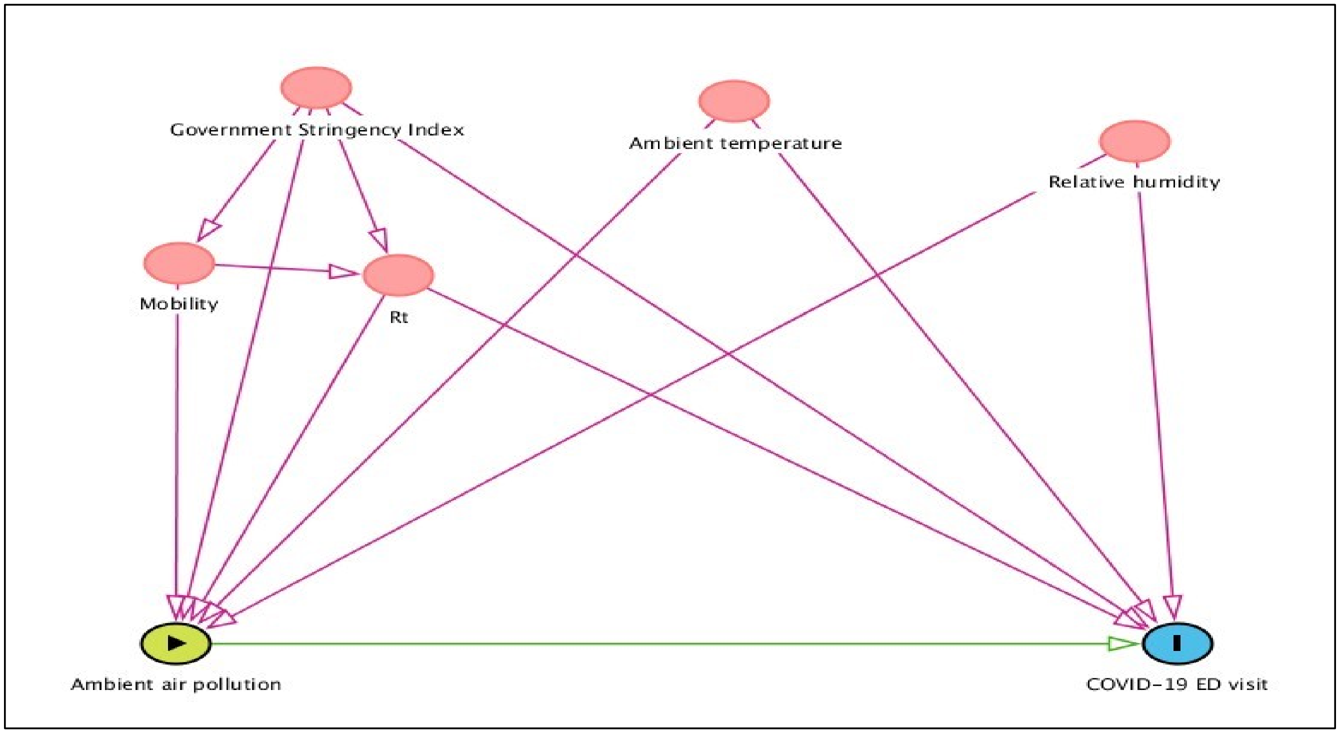

Supplement: Supplementary file 1 [file thoraxjnl-2021-217602supp001.pdf]
